# Concussion in Sports

**DOI:** 10.3390/jfmk4020037

**Published:** 2019-06-19

**Authors:** Giuseppe Musumeci, Silvia Ravalli, Angela Maria Amorini, Giuseppe Lazzarino

**Affiliations:** 1 Department of Biomedical and Biotechnological Sciences, Human Anatomy and Histology Section, School of Medicine, University of Catania, 95100 Catania, Italy; 2 Research Center on Motor Activities (CRAM), University of Catania, 95123 Catania, Italy; 3 Department of Biomedical and Biotechnological Sciences, Medical Biochemistry Section, University of Catania, 95100 Catania, Italy

**Keywords:** concussion, cerebral concussion, mild traumatic brain injury, head injury, injury, prevention

## Abstract

Concussion, a peculiar type of mild traumatic brain injury (mTBI), is an injury frequently encountered in various contact and noncontact sports, such as boxing, martial arts, American football, rugby, soccer, ice hockey, horse riding, and alpine skiing. Concussion occurs anytime external forces of specific intensities provoke acceleration–deceleration of the brain, and it is characterized by the rapid onset of short-lived impairment of neurologic functions, spontaneously resolving within weeks, persisting for longer times only in a small percentage of cases. A wide range of molecular alterations, including mitochondrial dysfunction, energy deficit, and gene and protein expression changes, is triggered by concussion and lasts longer than clinical symptoms. In recent years, concussion has become a primary issue of discussion among sports medicine professionals, athletes, media, and sports sponsors in relation to athletes’ return to play, after a concussion. Continued improvement in prevention and management of concussed athletes requires extensive research from different disciplines. Research work needs to focus on both prevention and management. Researchers and clinicians’ efforts should be dedicated to a better understanding of the molecular changes occurring in the post-concussed brain and to clearly define healing after concussion for a safe return of athletes to play. It is essential for sports medicine professionals to stay informed about the advances in understanding concussions and how to rehabilitate each single player who sustained a concussion.

## 1. Introduction

Concussion is defined as a traumatically induced transient disturbance of brain function occurring when external forces of different intensities (the most dangerous are rotational and translational), not necessarily acting directly on the head, provoke rapid acceleration–deceleration of the brain [1,2]. Incorrectly, the term concussion is interchangeably used with mild traumatic brain injuries (mTBIs), a neglected pathological state involving the overwhelming majority of the head-injured population treated in emergency departments in Europe and the USA [3]. It should however be recalled that whilst, by definition, all concussions are mTBIs, not all mTBIs are concussions [3]. It has been stated that concussive head injury could be typically recognized by a rapid onset of spontaneously resolving neurological impairment accompanied, at the molecular level, by alterations of various cellular functions rather than by structural damages. [2,4,5,6]. Athletes, particularly those who practice contact sports, are more prone than non-athletes to sustaining concussions. Risk of concussion is, for example, extremely frequent in full-contact sports such as boxing, martial arts, American football, ice hockey, Rugby Union, Rugby League, and Australian Rules football. Concussions in occasional contact sports, such as soccer and basket, are less frequent and in noncontact sports (biking, alpine skiing, tennis) are rare, but they occur as well. In this context, their consequences have been studied in tennis, suggesting not to underestimate this issue even in these kinds of sports [7]. Instead, equestrian sports have one of the highest rates of concussions in the world. Indeed, the majority of concussions and mTBIs are higher in horse racing and equestrian than in boxing or American football [8,9].

**Figure 1:**
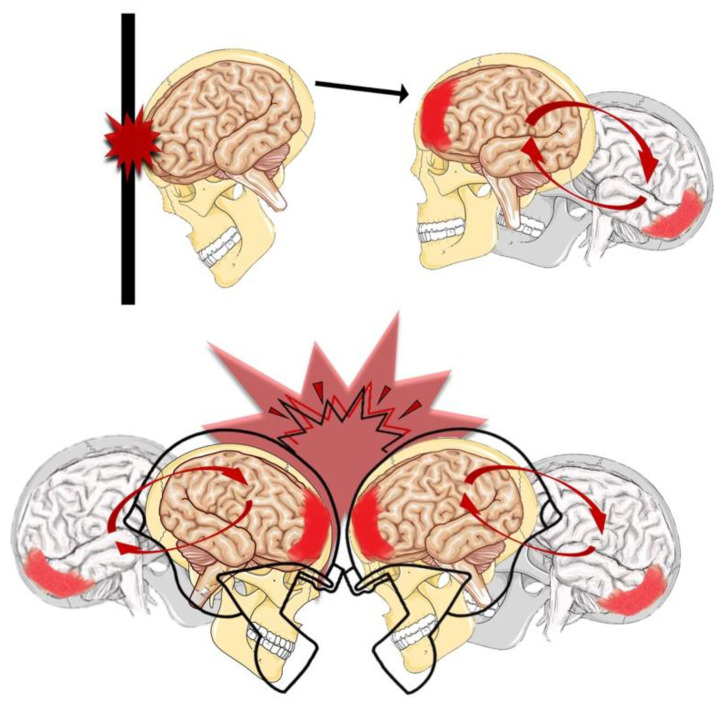
Concussion or traumatic brain injury (TBI) may alter brain functions. Concussion or traumatic brain injury could change the way the brain functions. This can lead to bruising and swelling of the brain, tearing of the blood vessels and injury to nerves, causing the concussion. The brain is made up of soft tissue and is protected by blood and spinal fluid. When the skull is jolted too fast or is impacted by something, the brain shifts and hits against the skull. Most concussions are mild and can be treated with appropriate care. However, left untreated, they can be deadly. The figure was designed by hand made as previously done in our studies [51,52].

For amateur and non-athletes, problems connected to concussion are the influence of postconcussive symptoms on the everyday life. For semiprofessional and professional athletes, problems connected to concussion concern the amount of time of rest before they are again allowed to play. Since athletes are at a higher risk of occurrence of concussive episodes, they are the population of choice with which to undertake trials aimed to study the pathobiology of concussion, to biologically grade concussion, to determine the best rehabilitation therapies during the recovery period, to evaluate potential pharmacological treatments, and to establish clear objective parameters for a safe return to play [6,10]. As previously said, it is possible to define a concussion as a traumatic insult capable of provoking an acceleration–deceleration phenomenon within the skull (Figure 1) [3,11]. Thanks to the increasing knowledge on concussion, two main potentially fatal risks, associated to repetitive concussions, have been highlighted: (i) second impact syndrome (SIS) [12], which may occasionally occur when a second concussion falls within a period of time named “window of metabolic brain vulnerability” [13,14,15]; and (ii) chronic traumatic encephalopathy (CTE), which may occur in retired athletes who sustained a high number of concussions (mainly unreported) in their career [16]. SIS suddenly develops, after the second impact, into an untreatable malignant brain edema [12]. CTE is a progressive neurodegenerative tauopathy causing dramatic loss of neurocognitive functions and that evolves into dementia [16,17]. Therefore, the withdrawal of high-profile professional athletes after concussion to avoid the risks of repetitive concussion contributed to increasing the awareness of the sports community to the importance of these injuries [18,19]. The aim of this review is to discuss some aspects concerning physiology, referred symptoms, present clinical approaches, and to underline the current unanswered questions, encouraging further research.

## 2. Neurobiological Considerations

Part of the energy associated to a concussive impact is transferred to the cerebral tissue, triggering complex pathophysiological molecular processes, also termed as the neurometabolic cascade of concussion [3,13,14]. A plethora of biochemical, metabolic, and molecular changes transiently, but profoundly, alter cerebral cell functions and homeostasis. Modifications in corticomotor excitability or inhibition, following the impact, suggest alterations in metabolic, ionic, and neurotransmitter processes [20]. A concussed brain is less responsive to usual neural activation, and prolonged dysfunction could be more evident in case of premature cognitive or physical activity stimulations [3]. This temporary window during which the brain is metabolically vulnerable, characterized by mitochondrial dysfunction [15,21], glucose dysmetabolism [22], and impairment of cellular energetic metabolism [15,21,23], lasts much longer than symptom disappearance and neuropsychological (NP) tests returning to baseline, and it is subjected to great individual variability [15,23,24]. Notwithstanding the widely diffused practice of assessing and monitoring concussion only using gross, non-objective neurocognitive and physical tests, the concept that concussion is mainly a molecular phenomenon that should be evaluated with appropriate methods capable of semiquantitatively measuring objective molecular parameters is rather unknown [15,23,24]. 

## 3. Postconcussive Symptomatology

Since most of the concussive-injured patients are asymptomatic, the more significant proportion of them receives no medical attention and remains unreported. Clinically, concussion is not necessarily complemented by loss of consciousness, and it is connected with various physical (headache, equilibrium, vision disturbances, and so on), cognitive (memory, concentration, and so on.), emotional (behavior), and sleep alterations [25]. Postconcussive symptoms may continue for weeks, affecting everyday life and, if present for months, they are referred as persistent postconcussive symptoms (PPCS) [26]. In addition, although postconcussive symptoms spontaneously resolve in the large majority of cases, numerous studies support that concussions pave the way for metabolic and molecular brain vulnerability, showing that a second blow, sustained before the brain gets the chance to fully recover, results in worsening of various metabolic functions within the cells [21,22,27] and in prolonged period of recovery after the last concussive episode [28,29].

## 4. Clinical Evaluation and Treatment

The current management of concussions is largely incomplete and requires further advancements, especially in clinical and diagnostic evaluation. Although in various sports disciplines, the perception of the concussion-associated risks led to increasing the number of diagnoses, currently, there are no validated indicators to make correct prognosis and, most importantly, athletes’ healing is determined on symptom disappearance, returning to baseline of neuropsychologic tests and physical exams, i.e., no assays to measure brain metabolism and functions are performed before the return of athletes to play. Since concussed athletes may rapidly become asymptomatic with no specific physical pain, it is not rare that they refuse medical evaluation and, consequently, treatment [30]. Hence, in light of the information obtained in various experimental studies [13,14,15,21,22,27], the transitory effects of a single brain injury on the individual player’s physical and mental status should more accurately be monitored. The ability to provide objective information (at the molecular/biochemical level) regarding the exact nature of the effects of concussion, both in the short term and over time, has been missing. 

### 4.1. Neuropsychological Tests

Despite these problems, NP tests are currently and widely used since they are more sensitive to indirect cognitive impairment than clinical exams (mainly based on self-reported symptoms) [19,30]. NP tests are generally aimed at evaluating a neutral amount of brain–behavior relationships, even though concussions may appropriately be managed without the use of NP testing. Paper and pencil NP tests are considered more complete, testing different domains but also assessing for circumstances other than concussion. This last possibility, together with the subjective interpretation of the results by the examiner, may significantly contribute to confusing the correct evaluation of concussion [31]. NP testings should be used only as part of a comprehensive concussion management strategy and should not be used separately [31]. The perfect timing, frequency, and type of NP testing have not yet been determined. In certain situations, properly administered and interpreted NP testing requires an additional value to assess cognitive function and recovery in the management of sports concussions. It is still unknown if the use of NP testing in the management of sports concussion helps in preventing repeated concussion, disastrous injury or long-term problems. Overall, NP evaluation is helpful in the postconcussion management of athletes with assiduous symptoms or difficult sequences [32], but it is absolutely useless in determining brain metabolic recovery after concussion.

### 4.2. Neuroimaging Techniques

Neuroanatomical lesions are not present in this type of mTBI, meaning that concussed patients are negative on classical imaging techniques such as CT scan and MRI. However, advanced neuroimaging techniques, such as functional MRI (fMRI) and diffusion tensor imaging (DTI), are useful tools with which to evidence concussion-associated metabolic and ultrastructural brain alterations [33,34]. Additionally, ^1^H-magnetic resonance spectroscopy (^1^H-MRS) is a valid neuroradiological approach allowing to measure brain specific metabolites, such as N-acetylaspartate (NAA), strictly connected to cerebral energy metabolism and mitochondrial functions [6,15,24,27]. Using this technique, it was demonstrated that brain metabolism of concussed athletes (with no previous history of concussion) is characterized by significant decrease in NAA, lasting much longer than symptom disappearance and spontaneously recovering about a month from impact [6,15,24]. Transcranial magnetic stimulation (TMS) is another valid approach for the analysis of concussion acknowledged by the latest consensus statement on concussion in sports [2]. TMS quantifies excitation and inhibition of the primary motor cortex, the spinal nerve roots, and the peripheral motor pathway and might be helpful in detecting neurophysiological changes in case of concussive injuries [35].

### 4.3. Medical Community Programs

The unpredictability of concussion and the high difficulty to identify cases in the general population have made large-scale studies in humans very limited. Lately, the research community has begun to implement programs for in-depth study of concussion in the sports arena [36], where concussed athletes can be identified and followed to assess neurocognitive short- and long-term effects and to consolidate the information currently obtained with the aforementioned advanced neuroimaging techniques. Today’s computer technology has allowed uniform documentation of injuries among multiple institutions. The ability to coordinate information from multiple sites, multiple professions, and a wide variety of athletes will provide the foundation for developing intervention programs for preventing and managing cases of concussion, for both athletes and non-athletes [37]. The success of these programs, aimed at minimizing the risks of concussion and identifying the best complementary approaches dedicated to the prognosis, monitoring, and recovery of concussed patients, is remarkably increasing. 

## 5. Future Insights

Future advancements will likely significantly ameliorate patient outcomes after sports-related concussion (SRC), highlight the optimal panel of diagnostic tests and assays for a safe return of athletes to play and, lastly, provide useful indications for identifying and appropriately managing those at risk for longer-term difficulties associated with repetitive head impact exposure. The wider public health implications of improving sports safety and encouraging developmentally suitable participation among youth and adolescents are particularly important long-term benefits of research in SRC [38]. Sports medicine physicians are frequently involved in the care of patients with sports concussion and should specially be trained to provide care along the continuum of sports concussion from the site where acute injury occurred to the return-to-play (RTP) decision moment [4,39]. In fact, recently, in the USA, in certain sports disciplines such as football, sports medicine physicians and specialists of concussion are even present on the playground just to assess on-the-field diagnosis of athletes suspected to have experienced a concussion [40]. SRC research has helped to grow consensus statements for clinical management through interdisciplinary efforts [2,3,41]. Therefore, involvement of neuroradiologists, biochemists, and molecular biologists will certainly allow improving the tools with which a concussed athlete is declared healed and ready to return to play. In this light, the role of neurorehabilitation in managing concussed athletes to improve recovery and increase the safety of the RTP moment is certainly crucial [42]. The present panorama of SRC research still requires large-scale investigations to better define the natural history of concussion and identify factors that should guide prevention, diagnosis, treatment, and return to play guidelines specific to each individual patient [43,44]. We currently know that the clinical and physiologic outcomes of a concussion are related but independent constructs deserving further scientific exploration. A series of studies have evidenced neurophysiologic changes, following a concussion, in the motor evoked potential waveform amplitude, in corticomotor conduction time, and in metabolic, ionic, and neurotransmitter (e.g., glutamate, GABA) processes [20]. This has sparked research incorporating advanced neuroimaging techniques, analysis of fluid biomarkers, and functional genomics and biomechanics, in addition to NP tests outcomes [23,24,33,34,44,45,46]. Furthermore, translational studies are constantly improving our understanding of optimal rehabilitation approaches, like subthreshold aerobic exercise [47,48,49,50], and have led to a shift from the earlier “complete rest” approach to the current gradual active management involvements after concussion [47]. 

## 6. Conclusions

Concussion is a disturbing and complicated problem in sports requiring a multidisciplinary approach for the diagnosis and management of athletes experiencing this peculiar type of brain injury. More important efforts are needed to educate athletes, parents, coaches, officials, school administrators, and healthcare providers and to develop concussion recognition, management and prevention. It is critical that athletes be aware of the importance of reporting every symptom or dynamics connected with concussions in order to help medical staff in detecting possible critical risks. Sports physicians and other health professionals should be trained in the evaluation and management of concussion, either because they have personal knowledge when it comes to specific athletes or because they are often in the best position to perform a correct diagnosis of an athlete suspected of having experienced concussion. Physicians should be prepared to provide counseling regarding potential long-term consequences of a concussion and recurrent concussions [53,54]. Primary prevention of some injuries may be possible with modification and enforcement of the rules and fair play. Helmets and mouth guards for some sports (football, lacrosse, hockey, soccer, rugby) are suited to preventing impact injuries (fracture, bleeding, laceration, and so on), but they have not been shown to reduce the incidence and severity of concussions. Secondary fundamental prevention may be possible through appropriate RTP management [47,48,49,50]. The RTP decision is a medical one. No athlete diagnosed for concussion should be allowed to RTP on the same match or while symptomatic, and the motto “if in doubt sit them out” should be applied worldwide in any sports discipline. Since main modifications of concussion occur at the molecular level [13,14,15,21,22,27], tests allowing to measure these parameters, such as advanced neuroradiological techniques (^1^H-MRS, fMRI, DTI, TMS), should mandatorily be included as useful and necessary tools to monitor recovery of post-concussed athletes. RTP should be recommended not only when symptoms clear off and NP tests return to baseline, but also when metabolic and molecular parameters return to pre-impact values. Appropriate combination of multiple diagnostic approaches should certainly be helpful in postconcussion management. Further studies are needed to consolidate the importance of advanced neuroimaging tools in the diagnosis, prognosis, and RTP decision of concussed athletes, to validate RTP guidelines, to improve identification of the minority proportion of patients having prolonged concussive symptoms, to reduce risks for SIS or CTE occurrence, as well as of other short-term or long-term complications.
